# Uric Acid

**Published:** 1906-09

**Authors:** 


URIC ACID. The Chemistry, Physiology and Pathology of Uric Acid, and the Physiologically Important Purin Bodies, with a Discussion of Metabolism in Gout. By Francis H. McCrudden, Laboratory of Physiological Chemistry, Harvard Medical School. Canvas, $3.00, net; paper, $2.50, net. Paul B. Hoeber, Medical Books, 69 E. 59th street, New York.

Although the metabolism of uric acid is by no means understood at the present moment, yet several researches have been carried out recently which settle many of the fundamental points in the theory of uric acid metabolism and gout. There are few subjects in medicine that have provoked such bitter controversies and wide range of theories as has uric acid. McCrudden has gone into the subject most deeply, and presents the material systematically, going from the simple facts of pure chemistry of Purin bodies to gradually more complex conditions of metabolism, so that by the time the section on metabolism in gout is reached, all the facts upon which different theories of gout are based have already been treated in their proper place. The contributions of all the leading workers in this field have been carefully reviewed, and a most elaborate bibliography is given. This is without doubt one of our most excellent treatises upon uric acid and gout.

